# A Systematic Review of Microbiota in Cirrhosis: A Change Towards a More Pathogenic Predisposition

**DOI:** 10.3390/ijms26020527

**Published:** 2025-01-09

**Authors:** Elias Xirouchakis, Alexandros Pelekanos, Spyridon Xirouchakis, Hariklia Kranidioti, Spilios Manolakopoulos

**Affiliations:** 1 Gastroenterology-Liver-Endoscopy Unit, 2nd Department of Internal Medicine, General Hospital of Athens “Hippocration”, National and Kapodistrian University of Athens, 115 27 Athens, Greece; alexander.pelekanos@gmail.com (A.P.); harakranidioti@yahoo.gr (H.K.); smanolak@med.uoa.gr (S.M.); 2 Department of Gastroenterology and Hepatology, Athens Medical, P. Faliron Hospital, 175 62 Athens, Greece; spyxirou@gmail.com; 3 Medical School, European University of Cyprus, 2404 Nicosia, Cyprus

**Keywords:** cirrhosis, dysbiosis, microbiota, hepatic encephalopathy, hepatocellular carcinoma, bacterial overgrowth

## Abstract

The microbiome of the human intestine is a regulator of health that modulates immune response and plays an important role in metabolism. The diversity, and abundance of microbiota communities in the gut have been shown to change in cirrhosis and its complications. We aimed to review the current knowledge regarding microbiota alterations in cirrhosis, its potential differences according to etiology, and its role in the development of cirrhosis complications. A systematic search of the online bibliographic database up to July 2024 was performed. Randomized controlled trials and observational and cohort studies that included a total or at least a cohort of cirrhotic adult patients were enlisted for data extraction and analysis. A total of 73 publications were included for data extraction. Alpha diversity was found to decrease in cirrhotic patients in 30/38 (78%) of the studies, while beta diversity in 20/22 (90%) presented significant differences between healthy and cirrhotic groups. Proteobacteria significantly increased in 20/27 (74%) studies, followed by Actinobacteria and Fusobacteria, while 22/25 (88%) studies found either a reduction in cirrhotic patients or increased abundance in healthy controls for Firmicutes and Bacteroidetes. The most abundant genera in hepatic encephalopathy groups were pathobionts such as Enterococcus and Streptococcus, followed by Vellionella and Escherichia. Heterogeneity was found among studies regarding Alpha diversity in hepatocellular carcinoma (HCC) as it was decreased in three studies, indifferent in five, and increased in three studies in comparison to cirrhotic non-HCC patients. The dysbiosis of the gut microbiota is associated with cirrhosis and the development of complications such as hepatic encephalopathy and hepatocellular carcinoma.

## 1. Introduction

The liver has multiple metabolic functions; situated between the gut and the rest of the human organism, it also plays an important role in immune-mediated defensive activities. It has been described as part of the gut–liver axis due to its significant first-pass effect over microbes, medical molecules, and toxic substances such as alcohol and food [1,2]. Many chronic liver diseases are developed once this important protective mechanism is damaged or saturated [1,3]. Additionally, several complications that appear in liver cirrhosis depend on these altered pathophysiological mechanisms [1].

The gut microbiome, which grows and resides in all parts of the gut, especially in the small intestine, exerts many effects that help or sometimes damage liver function, such as the metabolism of bile acids [4], alcohol [5], and immune tolerance [3,6]. Even though the liver has a high capacity for immune protection and metabolic reactions, these capabilities are not unlimited. Therefore, an intact barrier to control input is necessary [7]. This barrier is represented by the gut, which forms a complicated system built on different layers [1]. The main layers of this barrier are the mucus layer, the enteric cellular layer including enterocytes, goblet cells, tuft cells, and enterochromaffin cells, the rich immune system layer in between and under the enteric cells, and finally, the vascular system layer, which delivers all contents to the portal vein. In liver diseases, significant changes have been described in the production of mucins and short-chain fatty acids, both controlled by the presence of “healthy” commensal bacteria [1,6]. In addition, a reduction in the population of bacteria containing 7a glucuronidase changes the composition of secondary bile acids and induces reactions through the FXR receptor [4]. Finally, a leaky gut status is caused by microbiota changes, which produce an immune-mediated reaction that increases the turnover of enterocytes and alters the formation of proteins of the tight junctions [5,8]. These are probably the most important pathophysiologic mechanisms that have been described to date.

Dysbiosis refers to any persistent change or imbalance in the number, diversity, or abundance of microbial populations in the gut compared to a previously healthy state [9]. Dysbiosis is found in the early stages of many chronic liver diseases before and after the development of cirrhosis [10,11]. All microbiota changes represent an imbalance between protective bacteria like the ones included in the Firmicutes taxon and potentially pathogenic bacteria like Proteobacteria (*E. coli*, Klebsiella) [10]. In addition, in several instances during chronic liver diseases, certain microbial populations reach levels conventionally described as bacterial overgrowth [10,11,12,13,14]. In cirrhosis, a significant factor contributing to the development of bacterial overgrowth is small intestinal dysmotility, which seems to be reduced by portal hypertension [15,16].

Therefore, in the cirrhosis stage, especially when portal hypertension has been developed, the presence of dysbiosis with or without bacterial overgrowth and leaky gut can cause bacterial translocation and, consequently, septic episodes of bacterial peritonitis, variceal bleeding, and hepatic encephalopathy [1,8,10,12,16]. The purpose of this review is to collect the current knowledge regarding microbiota changes in cirrhosis, its differences based on etiology, and its role in the development of cirrhosis complications.

## 2. Methods

### 2.1. Data Identification

We searched the PUBMED database from 1970 to 14 July 2024 for publications written in the English language using text words including Gut Microbiota/Gut Flora/Gut Microbioma AND Cirrhosis. We manually searched for references from review articles and original studies.

### 2.2. Inclusion and Exclusion Criteria

Studies included for analysis needed to fulfill the following criteria: (i) published as a full paper; (ii) retrospective, prospective, or randomized controlled design; (iii) including in total or at least a cohort of cirrhotic patients (iv) no animal studies, and (v) involving a population aged over or equal to 18 years.

Studies were excluded if (i) they were analyzing or comparing results of a specific treatment on microbiota, (ii) included groups referring to transplanted patients, (iii) had no taxonomic analysis, (iv) reported microbiota other than the intestinal variety, and (v) focused on fungal or viral species.

### 2.3. Data Extraction and Methodological Assessment

**Figure 1:**
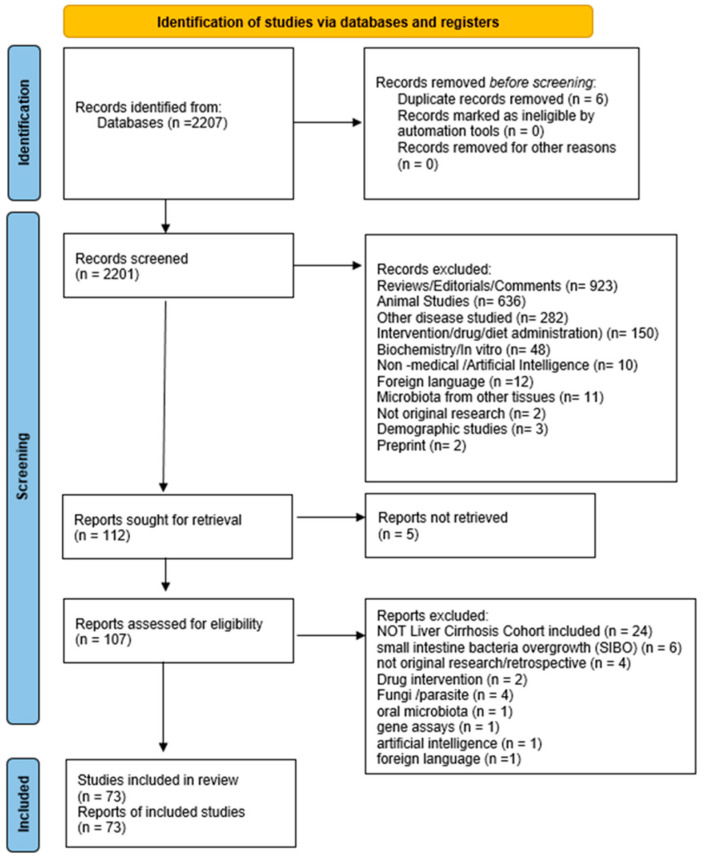
PRISMA flowchart of excluded and included studies for microbiota changes in cirrhosis.

This work was conducted with the PRISMA statement of Preferred Reporting Items for Systematic Reviews and Meta-Analyses [17] (Supplementary Table S1). The PRISMA flowchart is licensed under CC BY 4.0 (Figure 1). Data were extracted independently and tabulated using a predefined review form. Extracted data included publication information; study design; the time period that the study was performed in; the country where the study was performed; participant numbers; cirrhosis stage, the etiology and complications of groups; gut microbiota data collection, sequencing and analysis methods (e.g., sample type and mapping database used); and gut microbiota outcome data (e.g., specific taxa and diversity).

Thereafter, two authors were assigned for the quality assessment. The AMSTAR 2 tool was used to assess the quality of the studies (Supplementary Table S2). AMSTAR 2 did not involve a scoring system but rather a rating of the overall confidence of the results of the review ranging from high to critically low [18]. The decision to include or exclude a study was made before the review analysis was carried out and agreed upon consensus amongst the authors. In the present review, agreement between reviewers for the selection of articles for all analyses was 100%. The authors had no competing interests, and no funding was received for this review. The review was not registered.

### 2.4. Endpoints

Our endpoints for the present review were to assess the differences in alpha and beta diversity indices and to report taxonomic changes in microbiota in patients diagnosed with cirrhosis compared to healthy subjects or patients with chronic liver disease and no cirrhosis. Alpha diversity, as a measure of describing the richness and distribution of bacterial species in a single sample, and beta diversity, as a measure of the similarity between samples, were presented in most of the studies. We recorded results regarding diversity with the descriptors “increased”, “decreased”, or “not different” between study groups.

Bacteria were categorized into taxa according to their traits, such as morphology, and were classified into ranked groups labeled as phyla, class, order, family, genus, and species, in order of increasing affinity. Studies that were involved in this review reported taxa at various ranks that presented different abundance in the gut microbiota of those with cirrhosis. These results were accumulated and are presented in charts or tables.

A meta-analysis was not conducted due to the heterogeneity of the studies and the processing methodologies involved.

For further analysis, we also reported the following subgroups: (1) according to cirrhosis etiology (ALD, MASLD, HBV, etc.); (2) according to cirrhosis stage or complications (compensated vs. decompensated, hepatocellular carcinoma (HCC), hepatic encephalopathy (HE), or spontaneous bacterial peritonitis (SBP) and infections, or acute on chronic liver failure (ACLF); (3) according to the sample used (fecal samples, duodenal fluid, rectal swabs, or colon biopsies); (4) according to the test used (16S PCR, deep sequencing metagenomics, or other); and (5) according to geographical distribution. In addition, we checked for papers that reported microbiota in cirrhotic patients vs. non-cirrhotic patients of the same etiology.

## 3. Results and Discussion

The search strategy found 2207 references that were imported for screening (Figure 1).

Six duplicates were found and removed. The number of studies screened by title and abstract was 2201, and 2095 of them were excluded according to the aforementioned criteria. Of the remaining 122 articles, 5 were excluded due to no full-text retrieval. Of the 117 full-text articles screened for eligibility, 44 were excluded as 24 did not include a liver cirrhosis group, 4 were not original research, 10 did not report results on microbiota taxonomic differences (4 were about fungi or parasites and 6 reported only the presence of small intestinal bacterial overgrowth) and 6 did not meet the inclusion criteria (2 included drug interventions, 1 was about oral microbiota, 1 was on artificial intelligence practice, 1 was conducted as a gene assay, and 1 was in a non-English language).

Finally, we identified 73 studies that met inclusion criteria [12,15,19,20,21,22,23,24,25,26,27,28,29,30,31,32,33,34,35,36,37,38,39,40,41,42,43,44,45,46,47,48,49,50,51,52,53,54,55,56,57,58,59,60,61,62,63,64,65,66,67,68,69,70,71,72,73,74,75,76,77,78,79,80,81,82,83,84,85,86,87,88] (Figure 1). In these 73 studies, there was at least one cohort of patients diagnosed with cirrhosis (Supplementary Table S3). The diagnosis of cirrhosis was confirmed either histologically by liver biopsy or combinations of clinical, laboratory parameters, and/or imaging features (abdominal ultrasound/elastography).

In order to compare our findings, we also separately analyzed patients with decompensated cirrhosis and complications (Supplementary Table S3). Regarding the diagnosis of decompensation, this was confirmed by a history of clinical, endoscopic, and imaging features of ascites, HE, varices, and jaundice. Studies that solely included patients with cirrhosis and HE or HCC were analyzed independently (Supplementary Table S3) and were not included in the analysis of studies that compared compensated and decompensated patients. Studies that included arms of patients receiving antibiotics or immunomodulating medications were excluded.

### 3.1. Microbiota Diversity in Cirrhosis

**Figure 2:**
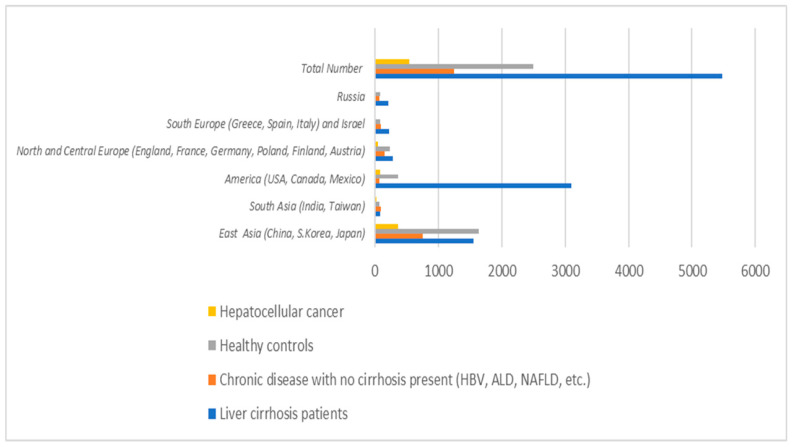
Patients included in the study and their relative groups of etiology according to geographical distribution.

To assess microbiota changes, we analyzed the data from 73 studies (Supplementary Table S3). A total number of 9.763 individuals was extracted. Of them, 2494 were healthy control groups, 5473 were patients with liver cirrhosis, 1246 were patients with chronic liver disease without cirrhosis, and 550 were patients with HCC. With regard to the geographical distribution (Supplementary Table S4), 33 studies were from East Asia (China 29, Japan 3, Southern Korea 1), 3 were from South Asia (Taiwan 2, India 1), 21 were from North or Central America (USA 19, Canada 1, Mexico 1), 6 were from North or Central Europe (Poland 1, UK 1, Germany 1, France 1, Finland 1, Austria 1), and 9 were from South or East Europe or Israel (Russia 4, Italy 2, Spain 1, Israel 1, Greece 1) (Figure 2).

Most studies used fecal samples to identify and quantify human gut microbiota (67/73 91.7%) (Supplementary Table S3). The remaining two studies used duodenal biopsies, another two used a combination of fecal samples and colon biopsies; and another study used a rectal swab. In addition, most studies (50/73, 68.4%) used 16s r-RNA sequencing as their method of detection, while fewer (8/73, 10.9%) appointed the Shotgun Metagenomics method, and (6/73, 8.2%) 16s r-DNA sequencing (Supplementary Table S3). PCR and bacterial cultures were the least used methods. Almost all studies investigated the entire human gut flora, while only one focused on specific species and their role in cirrhosis pathogenesis was included. This Chinese cohort analyzed the subspecies of Desulfovibrio, and the results showed a species-specific increase in cirrhotic patients with a significant difference in its trait, such as its desulfurization ability [28].

The diversity and composition data of the gut microbiota available were presented in different manners. In 13 studies, the results focused on the most enriched species detected, whereas 53 studies focused on the most abundant genera, families, or phyla, and 7 reported the most enriched bacterial groups. In total, 12 phyla, 47 families, and 118 genera were reported.

**Figure 3:**
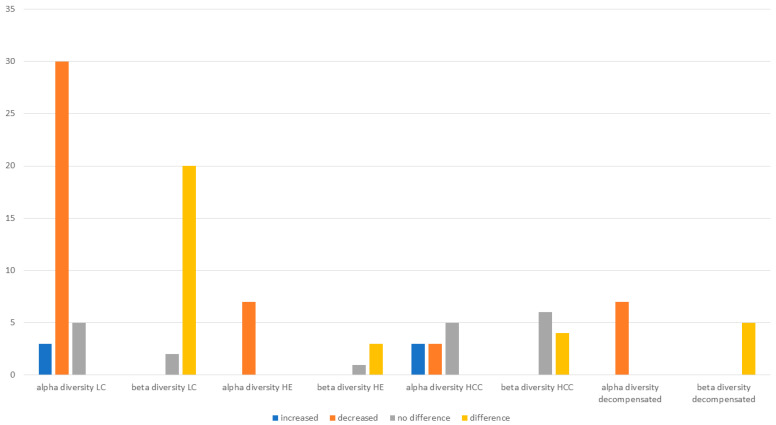
Results from studies reporting alpha and beta diversities of gut microbiota differences in liver cirrhosis (LC) patients vs. healthy controls (HC), cirrhotic patients with vs. without hepatic encephalopathy (HE), cirrhotic patients with vs. without hepatocellular cancer (HCC), and cirrhotic patients with vs. without decompensation.

Alpha diversity analysis in most studies was conducted using metrics such as Chao1, Ace, Shannon, and Simpson indices. Of the total 73 studies included, 53 (72%) reported results regarding alpha or beta diversity (Figure 3). Alpha diversity in healthy versus cirrhotic patients was presented in 38 studies. For cirrhosis without a specific etiology, there were 14/38 (37%) studies, while liver cirrhosis with a specific etiology (viral HBV or HVC, or metabolic NASH, or ALD) was the focus of 24 studies (63%). In 30 studies, a significant decrease in alpha diversity was observed (78%) in cirrhosis compared to healthy subjects: 5 showed no difference, and 3 noted an increase in liver cirrhosis groups (Figure 3). However, data from the latter three studies were collected from a rectal swab. The first study [45] indicated a possible difference in the composition of the rectal gut flora compared to the rest of the colon, the second included a very small Chinese cohort, possibly introducing bias [66], and the third study was from Taiwan comparing HBV-related cirrhotic patients with their non-cirrhotic counterparts [25]. In conclusion, despite a few discrepancies, most studies agree that a significant decrease in alpha diversity of the gut microbiota is associated with liver cirrhosis.

Regarding beta diversity, the most common metrics were Bray–Curtis dissimilarity and weighted and unweighted UniFrac. With the term cirrhosis without a specific etiology, there were 22 studies presenting results (Figure 3). In 20 of the 22 studies (90%), a significant difference between healthy and cirrhotic groups was shown, while in three studies, the results were opposing. This particular finding adds to the hypothesis that in cirrhosis, the intestinal microbiota undergoes significant changes in its composition that need to be revealed.

### 3.2. Gut Microbiota Composition in Liver Cirrhosis

**Figure 4:**
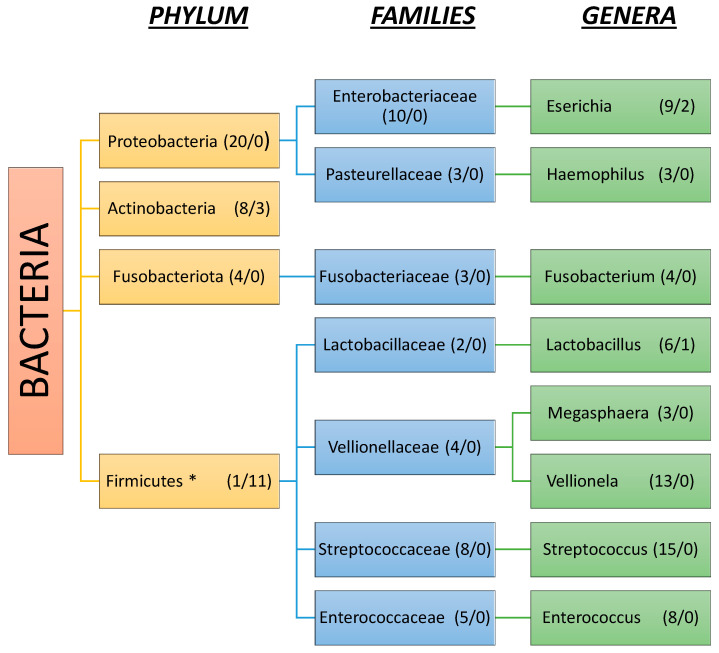
Gut microbiota enriched in liver cirrhosis. (The number of studies that support enrichment/the number of studies that support reduction). * Although the abundance of firmicutes is reduced in patients with liver cirrhosis compared to healthy subjects, some families and genera included in this phylum tend to be significantly enriched.

The human gut microbiota in cirrhosis is compromised by a plethora of species. Twenty-seven studies reported results on the phyla level, 21 on the family, and 44 on the genus level in patients with cirrhosis, while 25, 22, and 38, respectively, were reported in patients with no cirrhosis. At the phyla level, Proteobacteria seem to play the most important role in gut dysbiosis in cirrhosis, as they were significantly increased in 20/27 (74%) studies, followed by Actinobacteria and Fusobacteria (Figure 4).

**Figure 5:**
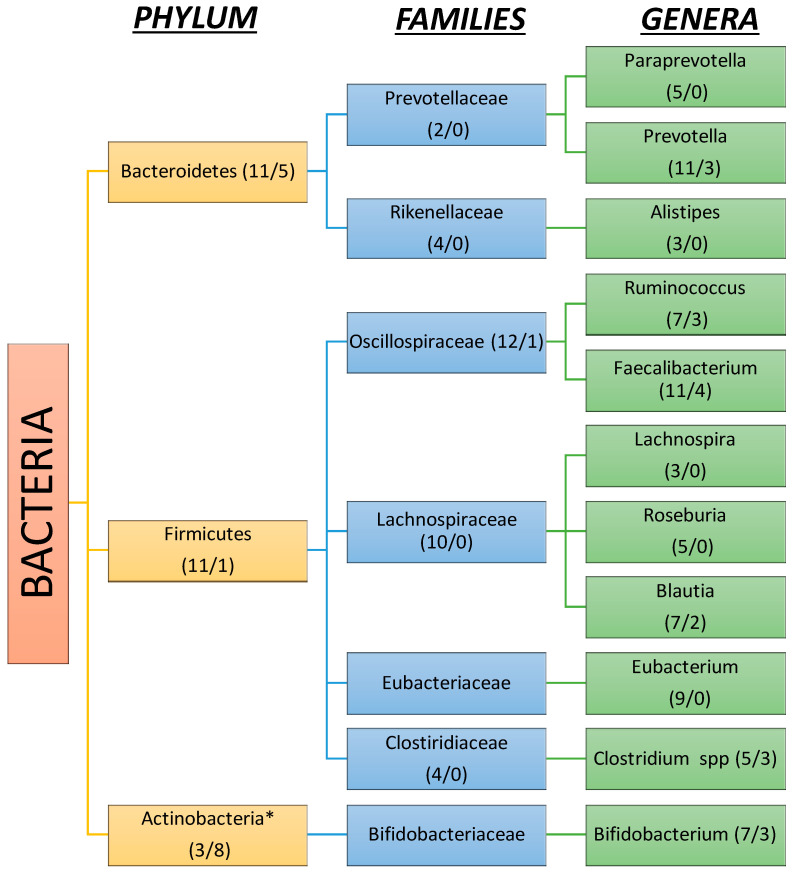
Gut microbiota decreased in liver cirrhosis vs. healthy subjects. (The number of studies that support reduction/the number of studies that support enrichment). * Although the abundance of Actinobacteria is increased in patients with liver cirrhosis compared to healthy subjects, Bifidobacterium tends to be more enriched in healthy vs. cirrhotic patients.

In contrast, 22/25 (88%) studies found either a reduction in cirrhotic patients or increased abundance in healthy controls for Firmicutes and Bacteroidetes (Figure 5). Notably, although most results agree on Proteobacteria and Firmicutes changes, there seems to be a discrepancy in Bacteroidetes as 11/25 (44%) studies reported an increase in healthy control versus cirrhotic groups while 5/27 (18%) reported the opposite. Another important aspect regarding Firmicutes is that at the level of sub-taxa, genera such as Streptococcus, Vellionella, and Enterococcus are considerably increased in numbers in cirrhotic patients than in non-cirrhotic subjects.

At the family level, changes found in liver cirrhosis consist of an increase in the abundance of Enterobacteriaceae, Pasteurellaceae, Fusobacteraceae, Lactobacillaceae, Vellionellaceae, Enterococcaceae and Streptococcaceae, and a decrease in Oscillospiraceae, Lachnospiraceae, Clostiridiaceae, Eubacteriaceae, Rikenellaceae, and Bifidobacteriaceae. In particular, Streptococcaceae and Enterobacteriaceae are the most commonly mentioned enriched bacteria, while Oscillospiraceae and Lachnospiraceae are the most decreased, respectively.

As far as genera are considered, we found decreases in autochthonous taxa such as Prevotella, Paraprevotella, Roseburia, Faecalibacterium, Eubacterium, and Ruminococcus. Otherwise, conflicting results were presented among studies regarding *Bifidobacterium*, *Clostiridium* spp., and *Bacteroides*, with most of them in favor of a decrease with increased abundance only evident in certain cases. A possible explanation for this heterogeneity is the fact that different etiologies of chronic liver diseases such as ALD, NAFLD, and HBV may influence microbiota even before progression into cirrhosis and remain thereafter. On the other hand, genera that are largely facultative anaerobes, such as Enterococcus, Escherichia, Klebsiella, Lactobacillus, Streptococcus, and Haemophilus increase in cirrhosis. Vellionella, an oral cavity bacterial species, is also significantly increased in cirrhotic patients. Finally, another important finding is that the *Rumminococcus gnavus* group has been linked to cirrhosis in at least three studies where most *Rumminococcus* spp. are significantly decreased in the cirrhotic gut environment [23,26,85].

### 3.3. Gut Microbiota Composition in Hepatic Encephalopathy

Hepatic encephalopathy (HE), a major complication of decompensated cirrhosis, is characterized by a broad spectrum of neuropsychiatric symptoms [89]. It can be classified as covert (Grade I) and subsequently minimal HE (recognized by specific cognitive tests), and overt, ranging from Grade II to IV exhibiting severe neuropsychiatric manifestations [90]. Most studies focused on minimal HE, using different scores for diagnosis, such as PHES, NCT-A, and DST. We found 16 studies in total that appointed results for HE either regarding taxonomy (5 on phyla, 7 on families, and 11 on genera) or diversity (7 on alpha and 4 on beta diversity). Alpha diversity in cirrhotic patients with HE was reported in 100% (7/7) of the studies to be decreased in comparison to cirrhotic patients without HE.

**Figure 6:**
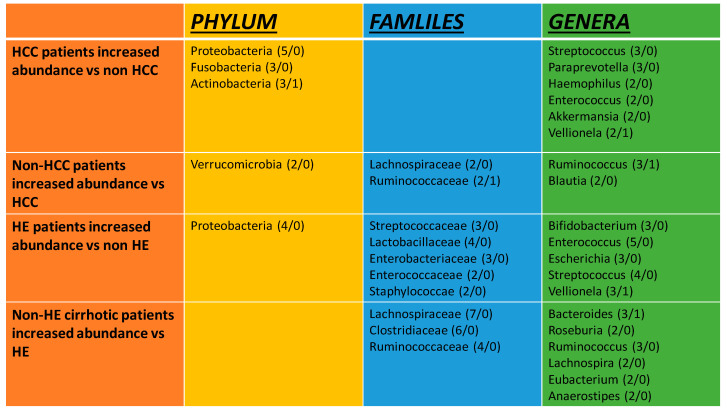
Comparison of hepatic encephalopathy and hepatocellular carcinoma as the most presented bacteria taxon in the studies. (The number of studies that show an increase/the number of studies that show no increase or decrease). Taxa that are reported in the results of at least two or more studies are presented in the graph.

The main phyla that were enriched in HE patients were Proteobacteria, whereas for families, there were Streptococcaceae, Lactobacillaceae, Enterobacteriaceae, Enterococcaceae, and Staphylococceae. The most abundant genera were pathobionts such as Enterococcus and Streptococcus, followed by Vellionella and Escherichia [20,26,41,43,51,54,65,75,77,86] (Figure 6). Notably, in a Chinese study, *Streptococcus salivarius* was strongly associated with the presence of HE in cirrhotic patients [77]. Another interesting finding was that the presence of Bifidobacterium, which is an autochthonous taxon with beneficial effects, was reported to be enriched in three studies [51,75,82]. A study from the USA associated Alcaligeneceae and Porphyromonadaceae with worse cognition [65]. In order to evaluate microbiota composition in MHE, one study demonstrated results based on three types of cognitive tests; although not completely matching, the results had similarities regarding bacterial groups that were enriched, such as bacteria from the Lactobacillaceae family [72].

On the contrary, some bacteria that were decreased in patients with HE compared to cirrhotic patients without encephalopathy were members from the families Lachnospiraceae, Clostridiaceae, Ruminococcaceae, such as Bacteroides, Roseburia, Ruminococcus, Lachnospira, and Eubacterium [20,26,41,43,51,60,65,72,75,77,80,81] (Figure 6).

### 3.4. Gut Microbiota Composition in Hepatocellular Carcinoma

Epidemiological studies have shown that 70% to 90% of hepatocellular carcinoma cases are found in patients with preceding cirrhosis or advanced fibrosis [91]. We identified 13 studies reporting microbiota alterations in patients with HCC (Figure 6). Alpha diversity showed a notable heterogeneity. It was found to be decreased in three studies [27,58,92], increased in three studies [40,59,74], and five of them [30,44,55,71,83] showed no differences compared to cirrhotic patients without HCC. These results may be derived from the difference in populations of gut microbiota between patients who develop HCC in the presence of cirrhosis as opposed to those without HCC, as some studies addressed [40,58]. Additionally, beta diversity showed no differences between groups in six studies [30,40,44,55,59,71], whereas four studies showed dissimilarity [27,46,58,92].

Considering gut flora composition, at the phyla level, changes include an increase in Proteobacteria, Fusobacteria, and Actinobacteria, and at the family level, some beneficial taxa like Lachnospiraceae and Rumminococcaceae belonging to the Firmicutes decrease. Genera that were proven to be enriched were Paraprevotella, Akkermansia, Haemophilus, and pathobionts such as Streptococcus and Enterococcus, while Ruminococcus, Blautia, and Bifidobacterium were decreased. Taken together, the above findings suggest a significant increase in pathogens like Proteobacteria and Streptococcaceae and, on the other hand, a decrease in beneficial bacteria such as those that reduce hydrophobic bile acid. As the role of pathogens in the development of HCC is not clear, a Polish study of patients with liver cirrhosis undergoing liver transplantation found a significant correlation between fecal *E. coli* and the presence of HCC [68]. In addition, while Lactobacillus is known to be beneficial in human homeostasis, as it belongs to a group of certain bacterial genera that are involved in bile acid deconjugation, oxidation/epimerization, and 7-dehydroxylation together with Bacteroides, Bifidobacterium, Ruminococcus, and Clostridium [93], a study from Germany on NASH associated the presence of HCC in cirrhotic patients with increased abundance in the Lactobacillus species [58] (Figure 6).

### 3.5. Gut Microbiota Composition in Other Complications

Few studies have assessed microbiota changes and other complications such as ascites, spontaneous bacterial peritonitis (SBP), infections, or ACLF [31,32,34,38,47,48,53,57,82,86]. Regarding microbiota changes during the passage from compensated to decompensated cirrhosis [31,36,40,42,55,56,57], or based on the Child–Pugh score [64,70], alpha diversity was found to be decreased in all studies (Figure 3). The most abundant bacterial groups were the Enterococcaceae, Streptococcace, and Peptostreptococcaceae families, while Enterococcus was the most prominent genus, followed by Streptococcus, Staphylococcus, Vellionella, and Lactobacillus. These findings follow the pattern already observed, with pathobionts increasing as liver disease progresses.

Clinically significant ascites are associated with an increase in Proteobacteria and a decrease in Actinobacteria and Bacteroidetes, as one study from Russia demonstrated [32]. Very few studies focused on SBP, including one from Russia that showed enrichment in Proteobacteria, particularly Gammaproteobacteria, including pathogens like *Klebsiella*, *Serratia*, *Acinetobacter*, and *Moraxella* spp. [31], and a study from India on cirrhotic patients with infections found an enrichment of Oxalobacteraceae, Neisseriaceae, and Anaerotruncus in SBP [34]. Gammaproteobacteria, Lactobacillaceae, and Enterobacteriaceae were found to be enriched in patients with concomitant infections such as pneumonia, bacteremia, and cholangitis but the results were sparse among different infections [34,38,65,86].

Finally, in ACLF, *Enterococcus* spp. was found to be the most prominent taxon [48,57], while in another study from the USA, Proteobacteria, Actionobacteria, and Cambylobacteracae were most abundant [82].

### 3.6. Gut Microbiota Assessment in Liver Cirrhosis Various Etiologies

Of the reviewed studies, 11 were conducted on HBV, 7 on ALD, and 6 on NAFLD populations. As already mentioned, alpha diversity is mostly decreased in cirrhotic patients, and this pattern shows no exception across different etiologies. Specifically, of the eight studies that were conducted on HBV cirrhotic patients reporting results on alpha diversity, six indicated a decrease [27,36,42,48,52,56,92], one indicated indifference [29], and one indicated an increase [25], while five reported differences in beta diversity versus non-cirrhotic controls. Regarding NAFLD, five reported decreased alpha diversity and a high beta diversity index [58,69,71,85,88]. In ALD, four studies presented decreased alpha diversity [40,49,59,61], while only two showed no significant difference [73,87].

The main phyla enriched were Actinobacteria and Proteobacteria in HBV cirrhotic populations presented in four and five studies, respectively; Actinobacteria, Proteobacteria, and Fusobacteria in ALD; and Proteobacteria mainly in NAFLD cirrhotic populations. The most prevalent families were Enterobacteriaceae in all etiologies [27,39,42,49,56,63,71,88] and Streptococcaceae in HBV populations [19,27,42,56]. Notably, Streptococcus and Vellionella species were presented in six [19,27,28,36,56,92] and five studies [25,27,56,62,92], respectively, and were the most increased genera in HBV cirrhotic populations, while Lactobaccilus was significantly increased in ALD groups [44,59,87].

In the present systematic review, we collected all published studies that identified the status of microbiota in cirrhosis. Our findings showed that cirrhotic patients have significant changes in both alpha and beta diversity compared to controls, which in most studies were healthy volunteers. Changes referred to all taxa of bacteria, namely phylum, families and genera. At the level of phylum, we uncovered conflicting results showing both an increase and decrease in abundance, but at a lower level, we found specific changes in families, and genera. We can conclude that at Proteobacteria phylum, Enterobacteriaceae and Pasteurellaceae families increase; at Firmicutes phylum, Lactobacillaceae Velionellaceae Streptococcacceae and Enterococcaceae increase, whilst Oscillospiraceae, Lachnospiraceae, Eubacteriaceae and Clostiridiaceae decrease. At Bacteriodetes phylum, Prevotelacceae and Rikenellaceae decrease, and at Actinobacteria, Bifidobacteriaceae decrease. Finally, at the Fusobacteria phylum, Fusobacteriaceae increase. Another important finding of this review is that as cirrhosis progresses to a decompensated stage and complications appear, there is a prominent change in both alpha and beta diversity as well. However, until now, the complication that has been most studied is hepatic encephalopathy. In hepatocellular carcinoma, studies show conflicting results regarding alpha and beta diversity.

A normal gut microbiota consists of six phyla, namely Proteobacteria, Firmicutes, Bacteroidetes, Actinobacteria, Fusobacteria, and Verrucomicrobia, with the phyla Bacteroidetes and Firmicutes representing more or less 90% of the population [94]. The normal distribution of gut microbiota in the intestinal lumen is associated with different pH values, motility, oxygen levels, and other conditions in the environment. The stomach is dominated by oral cavity bacteria such as Vellionella, Streptococcus, Prevotella, Rothia, and Haemophilus [95], and the duodenum is mostly facultatively anaerobic due to its hostile environment, while the jejunum contains both Gram-positive aerobic bacteria and facultative anaerobic like Lactobacillus, Enterococcus, and Streptococcus [96]. In the ileum, the main bulk consists of Enterococcus, Bacteroides, Lactobacillus, Clostridium, and Corynebacteria, while the cecum and the rest of the colon are harbored by anaerobic bacteria 100 to 1000 times more than aerobic bacteria. It is mainly inhabited by families such as Bacteroidaceae, Prevotellaceae, Rikenellaceae, Lachnospiraceae, and Ruminococcaceae, and regarding genera, the main species consist of Bacteroides, Lactobacillus, Bifidobacterium, and Clostridium. In addition, the colon hosts a plethora of pathogens such as *Campylobacter jejuni*, *Salmonella enterica*, *Escherichia coli* (*E. coli*), and *Bacteroides fragilis* [97]. In liver cirrhosis, several microbiota combinations appear specific when combined with other diseases like type 2 diabetes and inflammatory bowel disease [76]. Additionally, 54% of the new species that reside in the gut in cirrhosis are of buccal origin. Studies show that as cirrhosis progresses, significant microbiota and metabolic signatures are associated with the first decompensation event [38], and a significant change in the small intestinal environment indicates a shift in the disease state from compensated to decompensated with portal hypertension [22]. These microbiota changes in cirrhosis have produced some specific diagnostic models which were validated in recent years. These models provide both an approach to diagnosis and a method to evaluate new treatments. In 2014, the cirrhosis dysbiosis ratio (CDR) was found to be a useful quantitative tool for microbiome alterations in cirrhosis. A low ratio indicates dysbiosis [80], and it has already been used in studies evaluating treatment on microbiota with success [98]. Another diagnostic model presented in 2023 uses not only microbiota features but also metabolites. The seven microorganisms and two metabolites model uses the Subdoligranulum, Agathobacter, norank f Eubacterium coprostanoligenes group, Butyricicoccus, Lachnospiraceae UCG 004, and L-2,3-Dihydrodipicolinate, which are expected to be at high levels in cirrhosis patients and Blautia, Monoglobus, and 5-Acetamidovalerate at low levels [23]. Finally, a modified cirrhosis dysbiosis ratio (MDR): [Bacilli (%) + Proteobacteria (%)]/[Clostridia (%) + Bacteroidetes (%)] is able to indicate more severe states of cirrhosis in correlation to different levels of dysbiosis [47]. These are intended to replace the Firmicutes/Bacteroidetes (F/B) ratio and the Microbial Dysbiosis Index (MDI), which are not specific for cirrhosis [15].

Our systematic review summarizes many microbial changes in cirrhosis, and in most of them, a shift is realized towards an increase in potentially pathogenic or pathogenic bacteria and a significant decrease in commensal or beneficial bacteria. In fact, bacteria that play a role as metabolizers of bile salts, as producers of protective mucus, or as transformers of significant amino acids, lipids, and sugars are constantly reducing [5,8,12,63]. Many studies indicate not only that the presence of protective bacteria is reducing significantly but that the potentially pathogenic bacteria outnumber them, reaching the threshold of bacterial overgrowth, namely > 10^3^ cpu/mL [10,11,12,14]. This has been correlated with more advanced stages of cirrhosis, especially when significant portal hypertension is developed [22]. One study showed that the prevalence of SIBO is significantly higher in liver cirrhosis than in non-cirrhosis patients. Additionally, even in non-cirrhosis patients, when SIBO is present, this is correlated with irreversible advanced liver disease [99]. Another study showed that SIBO derived from hydrogen-producing bacteria is more important than SIBO from methane producers in the development of hepatic encephalopathy [100]. In addition, a study published by our group found a significant correlation between SIBO with the Child–Pugh score and significant motility changes in cirrhosis [16]. And this was in line with another study that showed that microbiota changes depend on small intestinal transit alterations found in cirrhosis with portal hypertension [15]. The last two studies also showed that in the presence of decompensation episodes with significant portal hypertension, pharmaceutical interventions that reduce SIBO, like rifaximin, or increase small intestinal motility, like propranolol, prove efficient [16,100]. Finally, the use of probiotics, mostly with multi-strain formulae, has been assessed in a recent systematic review, and the meta-analysis of 22 studies involving cirrhotic patients was conducted, 5 of them including patients with hepatic encephalopathy [101]. Regardless of several limitations reported by the authors, there seems to be a tendency to improve liver function, the levels of endotoxin, and inflammatory cytokines and to reduce episodes of hepatic encephalopathy when using probiotics. However, larger studies are needed to better define the outcomes and make solid recommendations given the increased cost. Taken together, the current knowledge described in this systematic review provides evidence for clinical implications regarding specific dysbiosis scores for cirrhosis as information that can be used for the correct use of probiotics, antibiotics, and targeted therapies like motility agents.

This systematic review has some limitations. Firstly, studies originate from different areas of the world with different dietary habits, which may have influenced the heterogeneity of the results. Additionally, some changes may be due to the differences in the etiology of cirrhosis or pharmacologic interventions in decompensated cirrhosis, such as the previous use of antibiotics and gastric antisecretory medications. Another possible limitation arises from the fact that nearly most of the studies analyzed microbiota on the stools provided, while the alternative of duodenal biopsies or duodenal aspirates may have proven a different biological diversity as other studies on microbiota have indicated [102]. Finally, 23 of the 73 studies included small samples of less than 50 patients, which may decrease the validity of these results.

## 4. Conclusions

In conclusion, dysbiosis of the gut microbiota is strongly associated with cirrhosis and its complications, such as hepatic encephalopathy and HCC. Studies conducted on the microbiome structure showed some heterogeneity in the results. Despite this, changes in alpha diversity and, more specifically, the increase in the abundance of pathobionts such as Enterococcus and Streptococcus and the decrease in species that belong to bacteria families that play a significant role in gut homeostasis including Lachnospiracaceae and Oscillospiracaceae were all linked with liver cirrhosis progression. These changes in microbiota composition directly and indirectly affect the pathophysiology of cirrhosis through various mechanisms, including chronic inflammation, changes in metabolism, and increased intestinal permeability. More comprehensive, large-scale studies are needed to investigate the role of microbiota in specific complications, including ascites, spontaneous bacterial peritonitis, and ACLF, as current knowledge lacks sufficient evidence on this matter.

## Data Availability

In the present study no new data were created.

## Supplementary Materials

The following supporting information can be downloaded at https://www.mdpi.com/article/10.3390/ijms26020527/s1. Reference [103] is cited in the Supplementary Materials.

## References

[1] Albillos A., de Gottardi A., Rescigno M. (2020). The gut-liver axis in liver disease: Pathophysiological basis for therapy. J. Hepatol..

[2] Arab J.P., Martin-Mateos R.M., Shah V.H. (2018). Gut-liver axis, cirrhosis and portal hypertension: The chicken and the egg. Hepatol. Int..

[3] Xirouchakis E., Manousou P., Tsartsali L., Georgopoulos S.D., Burroughs A.K. (2009). Insights into the pathogenesis of NAFLD: The role of metabolic and pro-inflammatory mediators. Ann. Gastroenterol..

[4] Collins S.L., Stine J.G., Bisanz J.E., Okafor C.D., Patterson A.D. (2023). Bile acids and the gut microbiota: Metabolic interactions and impacts on disease. Nat. Rev. Microbiol..

[5] Meroni M., Longo M., Dongiovanni P. (2019). Alcohol or Gut Microbiota: Who Is the Guilty?. Int. J. Mol. Sci..

[6] Park J.W., Kim S.E., Lee N.Y., Kim J.H., Jung J.H., Jang M.K., Park S.H., Lee M.S., Kim D.J., Kim H.S. (2021). Role of Microbiota-Derived Metabolites in Alcoholic and Non-Alcoholic Fatty Liver Diseases. Int. J. Mol. Sci..

[7] Jenne C.N., Kubes P. (2013). Immune surveillance by the liver. Nat. Immunol..

[8] Forlano R., Martinez-Gili L., Takis P., Miguens-Blanco J., Liu T., Triantafyllou E., Skinner C., Loomba R., Thursz M., Marchesi J.R. (2024). Disruption of gut barrier integrity and host-microbiome interactions underlie MASLD severity in patients with type-2 diabetes mellitus. Gut Microbes.

[9] Marchesi J.R., Adams D.H., Fava F., Hermes G.D., Hirschfield G.M., Hold G., Quraishi M.N., Kinross J., Smidt H., Tuohy K.M. (2016). The gut microbiota and host health: A new clinical frontier. Gut.

[10] Acharya C., Bajaj J.S. (2019). Altered Microbiome in Patients with Cirrhosis and Complications. Clin. Gastroenterol. Hepatol..

[11] Maslennikov R., Ivashkin V., Efremova I., Poluektova E., Kudryavtseva A., Krasnov G. (2022). Gut dysbiosis and small intestinal bacterial overgrowth as independent forms of gut microbiota disorders in cirrhosis. World J. Gastroenterol..

[12] Gkolfakis P., Tziatzios G., Leite G., Papanikolaou I.S., Xirouchakis E., Panayiotides I.G., Karageorgos A., Millan M.J., Mathur R., Weitsman S. (2023). Prevalence of Small Intestinal Bacterial Overgrowth Syndrome in Patients with Non-Alcoholic Fatty Liver Disease/Non-Alcoholic Steatohepatitis: A Cross-Sectional Study. Microorganisms.

[13] Efremova I., Maslennikov R., Alieva A., Poluektova E., Ivashkin V. (2023). Small Intestinal Bacterial Overgrowth Is Associated with Poor Prognosis in Cirrhosis. Microorganisms.

[14] Fitriakusumah Y., Lesmana C.R.A., Bastian W.P., Jasirwan C.O.M., Hasan I., Simadibrata M., Kurniawan J., Sulaiman A.S., Gani R.A. (2019). The role of Small Intestinal Bacterial Overgrowth (SIBO) in Non-alcoholic Fatty Liver Disease (NAFLD) patients evaluated using Controlled Attenuation Parameter (CAP) Transient Elastography (TE): A tertiary referral center experience. BMC Gastroenterol..

[15] Liu Y., Jin Y., Li J., Zhao L., Li Z., Xu J., Zhao F., Feng J., Chen H., Fang C. (2018). Small Bowel Transit and Altered Gut Microbiota in Patients with Liver Cirrhosis. Front. Physiol..

[16] Xirouchakis E., Kranidioti H., Hadziyanni E., Kourikou A., Reppas C., Vertzoni M., Papadopoulos N., Deutsch M., Papatheodoridis G., Manolakopoulos S. (2024). The effect of propranolol on gastrointestinal motility and permeability in patients with cirrhosis and significant portal hypertension. BMC Gastroenterol..

[17] Moher D., Liberati A., Tetzlaff J., Altman D.G., Group P. (2009). Preferred reporting items for systematic reviews and meta-analyses: The PRISMA statement. Ann. Intern. Med..

[18] Shea B.J., Reeves B.C., Wells G., Thuku M., Hamel C., Moran J., Moher D., Tugwell P., Welch V., Kristjansson E. (2017). AMSTAR 2: A critical appraisal tool for systematic reviews that include randomised or non-randomised studies of healthcare interventions, or both. BMJ.

[19] Yang J., He Q., Lu F., Chen K., Ni Z., Wang H., Zhou C., Zhang Y., Chen B., Bo Z. (2023). A distinct microbiota signature precedes the clinical diagnosis of hepatocellular carcinoma. Gut Microbes.

[20] Jinato T., Sikaroodi M., Fagan A., Sterling R.K., Lee H., Puri P., Davis B.C., Fuchs M., Gavis E., Gillevet P.M. (2023). Alterations in gut virome are associated with cognitive function and minimal hepatic encephalopathy cross-sectionally and longitudinally in cirrhosis. Gut Microbes.

[21] Wang Q., Tang X., Qiao W., Sun L., Shi H., Chen D., Xu B., Liu Y., Zhao J., Huang C. (2024). Machine learning-based characterization of the gut microbiome associated with the progression of primary biliary cholangitis to cirrhosis. Microbes Infect..

[22] Efremova I., Maslennikov R., Poluektova E., Zharkova M., Kudryavtseva A., Krasnov G., Fedorova M., Shirokova E., Kozlov E., Levshina A. (2023). Gut Dysbiosis and Hemodynamic Changes as Links of the Pathogenesis of Complications of Cirrhosis. Microorganisms.

[23] Chen Y., Chen S., Xu C., Yu L., Chu S., Bao J., Wang J., Wang J. (2023). Identification of Diagnostic Biomarkers for Compensatory Liver Cirrhosis Based on Gut Microbiota and Urine Metabolomics Analyses. Mol. Biotechnol..

[24] Aliwa B., Horvath A., Traub J., Feldbacher N., Habisch H., Fauler G., Madl T., Stadlbauer V. (2023). Altered gut microbiome, bile acid composition and metabolome in sarcopenia in liver cirrhosis. J. Cachexia Sarcopenia Muscle.

[25] Lin M.J., Su T.H., Chen C.C., Wu W.K., Hsu S.J., Tseng T.C., Liao S.H., Hong C.M., Yang H.C., Liu C.J. (2023). Diversity and composition of gut microbiota in healthy individuals and patients at different stages of hepatitis B virus-related liver disease. Gut Pathog..

[26] Wang Q., Chen C., Zuo S., Cao K., Li H. (2023). Integrative analysis of the gut microbiota and faecal and serum short-chain fatty acids and tryptophan metabolites in patients with cirrhosis and hepatic encephalopathy. J. Transl. Med..

[27] Yan F., Zhang Q., Shi K., Zhang Y., Zhu B., Bi Y., Wang X. (2023). Gut microbiota dysbiosis with hepatitis B virus liver disease and association with immune response. Front. Cell. Infect. Microbiol..

[28] Lu G., Zhang Y., Ren Y., Shi J.S., Xu Z.H., Geng Y. (2023). Diversity and Comparison of Intestinal Desulfovibrio in Patients with Liver Cirrhosis and Healthy People. Microorganisms.

[29] Shen Y., Wu S.D., Chen Y., Li X.Y., Zhu Q., Nakayama K., Zhang W.Q., Weng C.Z., Zhang J., Wang H.K. (2023). Alterations in gut microbiome and metabolomics in chronic hepatitis B infection-associated liver disease and their impact on peripheral immune response. Gut Microbes.

[30] Lai M.W., Chu Y.D., Hsu C.W., Chen Y.C., Liang K.H., Yeh C.T. (2022). Multi-Omics Analyses Identify Signatures in Patients with Liver Cirrhosis and Hepatocellular Carcinoma. Cancers.

[31] Zhou Z., Lv H., Lv J., Shi Y., Huang H., Chen L., Shi D. (2022). Alterations of gut microbiota in cirrhotic patients with spontaneous bacterial peritonitis: A distinctive diagnostic feature. Front. Cell. Infect. Microbiol..

[32] Maslennikov R., Ivashkin V., Alieva A., Poluektova E., Kudryavtseva A., Krasnov G., Zharkova M., Zharikov Y. (2022). Gut dysbiosis and body composition in cirrhosis. World J. Hepatol..

[33] Hua X., Feng H. (2022). Changes in intestinal microbiota of HBV-associated liver cirrhosis with/without hepatic encephalopathy. Medicine.

[34] Philips C.A., Ahamed R., Abduljaleel J.K.P., Rajesh S., Augustine P. (2023). Identification and Analysis of Gut Microbiota and Functional Metabolism in Decompensated Cirrhosis with Infection. J. Clin. Transl. Hepatol..

[35] Bajaj J.S., Pena-Rodriguez M., La Reau A., Phillips W., Fuchs M., Davis B.C., Sterling R.K., Sikaroodi M., Fagan A., Shamsaddini A. (2023). Longitudinal transkingdom gut microbial approach towards decompensation in outpatients with cirrhosis. Gut.

[36] Sun X., Chi X., Zhao Y., Liu S., Xing H. (2022). Characteristics and Clinical Significance of Intestinal Microbiota in Patients with Chronic Hepatitis B Cirrhosis and Type 2 Diabetes Mellitus. J. Diabetes Res..

[37] Ullah N., Kakakhel M.A., Khan I., Gul Hilal M., Lajia Z., Bai Y., Sajjad W., Yuxi L., Ullah H., Almohaimeed H.M. (2022). Structural and compositional segregation of the gut microbiota in HCV and liver cirrhotic patients: A clinical pilot study. Microb. Pathog..

[38] Bajaj J.S., Reddy K.R., Tandon P., Garcia-Tsao G., Kamath P.S., O’Leary J.G., Wong F., Lai J., Vargas H., Thuluvath P.J. (2022). Association of serum metabolites and gut microbiota at hospital admission with nosocomial infection development in patients with cirrhosis. Liver Transpl..

[39] Baltazar-Diaz T.A., Gonzalez-Hernandez L.A., Aldana-Ledesma J.M., Pena-Rodriguez M., Vega-Magana A.N., Zepeda-Morales A.S.M., Lopez-Roa R.I., Del Toro-Arreola S., Martinez-Lopez E., Salazar-Montes A.M. (2022). Escherichia/Shigella, SCFAs, and Metabolic Pathways-The Triad That Orchestrates Intestinal Dysbiosis in Patients with Decompensated Alcoholic Cirrhosis from Western Mexico. Microorganisms.

[40] Zheng R., Wang G., Pang Z., Ran N., Gu Y., Guan X., Yuan Y., Zuo X., Pan H., Zheng J. (2020). Liver cirrhosis contributes to the disorder of gut microbiota in patients with hepatocellular carcinoma. Cancer Med..

[41] Bajaj J.S., Fagan A., McGeorge S., Sterling R.K., Rogal S., Sikaroodi M., Gillevet P.M. (2022). Area Deprivation Index and Gut-Brain Axis in Cirrhosis. Clin. Transl. Gastroenterol..

[42] Shu W., Shanjian C., Jinpiao L., Qishui O. (2022). Gut microbiota dysbiosis in patients with hepatitis B virus-related cirrhosis. Ann. Hepatol..

[43] Lin Y., Yan G., Feng F., Wang M., Long F. (2022). Characterization of intestinal microbiota and serum metabolites in patients with mild hepatic encephalopathy. Open Life Sci..

[44] Dong T.S., Jacobs J.P., Agopian V., Pisegna J.R., Ayoub W., Durazo F., Enayati P., Sundaram V., Benhammou J.N., Noureddin M. (2022). Duodenal Microbiome and Serum Metabolites Predict Hepatocellular Carcinoma in a Multicenter Cohort of Patients with Cirrhosis. Dig. Dis. Sci..

[45] Shen T.D., Daniel S.G., Patel S., Kaplan E., Phung L., Lemelle-Thomas K., Chau L., Herman L., Trisolini C., Stonelake A. (2021). The Mucosally-Adherent Rectal Microbiota Contains Features Unique to Alcohol-Related Cirrhosis. Gut Microbes.

[46] Chen T., Ding R., Chen X., Lu Y., Shi J., Lu Y., Tang B., Zhang W., Ye C., Yuan M. (2021). Firmicutes and Blautia in gut microbiota lessened in chronic liver diseases and hepatocellular carcinoma patients: A pilot study. Bioengineered.

[47] Maslennikov R., Ivashkin V., Efremova I., Alieva A., Kashuh E., Tsvetaeva E., Poluektova E., Shirokova E., Ivashkin K. (2021). Gut dysbiosis is associated with poorer long-term prognosis in cirrhosis. World J. Hepatol..

[48] Wang K., Zhang Z., Mo Z.S., Yang X.H., Lin B.L., Peng L., Xu Y., Lei C.Y., Zhuang X.D., Lu L. (2021). Gut microbiota as prognosis markers for patients with HBV-related acute-on-chronic liver failure. Gut Microbes.

[49] Zhong X., Cui P., Jiang J., Ning C., Liang B., Zhou J., Tian L., Zhang Y., Lei T., Zuo T. (2021). Streptococcus, the Predominant Bacterium to Predict the Severity of Liver Injury in Alcoholic Liver Disease. Front. Cell. Infect. Microbiol..

[50] Gui Q.F., Jin H.L., Zhu F., Lu H.F., Zhang Q., Xu J., Yang Y.M., Xiao C. (2021). Gut microbiota signatures in Schistosoma japonicum infection-induced liver cirrhosis patients: A case-control study. Infect. Dis. Poverty.

[51] Bloom P.P., Luevano J.M., Miller K.J., Chung R.T. (2021). Deep stool microbiome analysis in cirrhosis reveals an association between short-chain fatty acids and hepatic encephalopathy. Ann. Hepatol..

[52] Tang Y., Zhou H., Xiang Y., Cui F. (2021). The diagnostic potential of gut microbiome for early hepatitis B virus-related hepatocellular carcinoma. Eur. J. Gastroenterol. Hepatol..

[53] Yokoyama K., Tsuchiya N., Yamauchi R., Miyayama T., Uchida Y., Shibata K., Fukuda H., Umeda K., Takata K., Tanaka T. (2020). Exploratory Research on the Relationship between Human Gut Microbiota and Portal Hypertension. Intern. Med..

[54] Zha H., Chen Y., Wu J., Chang K., Lu Y., Zhang H., Xie J., Wang Q., Tang R., Li L. (2020). Characteristics of three microbial colonization states in the duodenum of the cirrhotic patients. Future Microbiol..

[55] Lapidot Y., Amir A., Nosenko R., Uzan-Yulzari A., Veitsman E., Cohen-Ezra O., Davidov Y., Weiss P., Bradichevski T., Segev S. (2020). Alterations in the Gut Microbiome in the Progression of Cirrhosis to Hepatocellular Carcinoma. mSystems.

[56] Chen Z., Xie Y., Zhou F., Zhang B., Wu J., Yang L., Xu S., Stedtfeld R., Chen Q., Liu J. (2020). Featured Gut Microbiomes Associated with the Progression of Chronic Hepatitis B Disease. Front. Microbiol..

[57] Sole C., Guilly S., Da Silva K., Llopis M., Le-Chatelier E., Huelin P., Carol M., Moreira R., Fabrellas N., De Prada G. (2021). Alterations in Gut Microbiome in Cirrhosis as Assessed by Quantitative Metagenomics: Relationship with Acute-on-Chronic Liver Failure and Prognosis. Gastroenterology.

[58] Sydor S., Best J., Messerschmidt I., Manka P., Vilchez-Vargas R., Brodesser S., Lucas C., Wegehaupt A., Wenning C., Assmuth S. (2020). Altered Microbiota Diversity and Bile Acid Signaling in Cirrhotic and Noncirrhotic NASH-HCC. Clin. Transl. Gastroenterol..

[59] Seok J., Suk K.T. (2020). Gut-microbiome Taxonomic Profiling as Non-invasive Biomarkers for the Early Detection of Alcoholic Hepatocellular Carcinoma. J. Liver Cancer.

[60] Haraguchi M., Miuma S., Masumoto H., Ichikawa T., Kanda Y., Sasaki R., Fukushima M., Miyaaki H., Taura N., Nakao K. (2019). Bacteroides in colonic mucosa-associated microbiota affects the development of minimal hepatic encephalopathy in patients with cirrhosis. Hepatol. Int..

[61] Addolorato G., Ponziani F.R., Dionisi T., Mosoni C., Vassallo G.A., Sestito L., Petito V., Picca A., Marzetti E., Tarli C. (2020). Gut microbiota compositional and functional fingerprint in patients with alcohol use disorder and alcohol-associated liver disease. Liver Int..

[62] Deng Y.D., Peng X.B., Zhao R.R., Ma C.Q., Li J.N., Yao L.Q. (2019). The intestinal microbial community dissimilarity in hepatitis B virus-related liver cirrhosis patients with and without at alcohol consumption. Gut Pathog..

[63] Chen Y., Yang F., Lu H., Wang B., Chen Y., Lei D., Wang Y., Zhu B., Li L. (2011). Characterization of fecal microbial communities in patients with liver cirrhosis. Hepatology.

[64] Wei X., Jiang S., Zhao X., Li H., Lin W., Li B., Lu J., Sun Y., Yuan J. (2016). Community-Metabolome Correlations of Gut Microbiota from Child-Turcotte-Pugh of A and B Patients. Front. Microbiol..

[65] Bajaj J.S., Ridlon J.M., Hylemon P.B., Thacker L.R., Heuman D.M., Smith S., Sikaroodi M., Gillevet P.M. (2012). Linkage of gut microbiome with cognition in hepatic encephalopathy. Am. J. Physiol. Gastrointest. Liver Physiol..

[66] Liu J., Wu D., Ahmed A., Li X., Ma Y., Tang L., Mo D., Ma Y., Xin Y. (2012). Comparison of the gut microbe profiles and numbers between patients with liver cirrhosis and healthy individuals. Curr. Microbiol..

[67] Zhang L., Wu Y.N., Chen T., Ren C.H., Li X., Liu G.X. (2019). Relationship between intestinal microbial dysbiosis and primary liver cancer. Hepatobiliary Pancreat. Dis. Int..

[68] Grat M., Wronka K.M., Krasnodebski M., Masior L., Lewandowski Z., Kosinska I., Grat K., Stypulkowski J., Rejowski S., Wasilewicz M. (2016). Profile of Gut Microbiota Associated with the Presence of Hepatocellular Cancer in Patients with Liver Cirrhosis. Transplant. Proc..

[69] Astbury S., Atallah E., Vijay A., Aithal G.P., Grove J.I., Valdes A.M. (2020). Lower gut microbiome diversity and higher abundance of proinflammatory genus Collinsella are associated with biopsy-proven nonalcoholic steatohepatitis. Gut Microbes.

[70] Jin M., Kalainy S., Baskota N., Chiang D., Deehan E.C., McDougall C., Tandon P., Martinez I., Cervera C., Walter J. (2019). Faecal microbiota from patients with cirrhosis has a low capacity to ferment non-digestible carbohydrates into short-chain fatty acids. Liver Int..

[71] Ponziani F.R., Bhoori S., Castelli C., Putignani L., Rivoltini L., Del Chierico F., Sanguinetti M., Morelli D., Paroni Sterbini F., Petito V. (2019). Hepatocellular Carcinoma Is Associated with Gut Microbiota Profile and Inflammation in Nonalcoholic Fatty Liver Disease. Hepatology.

[72] Bajaj J.S., Fagan A., White M.B., Wade J.B., Hylemon P.B., Heuman D.M., Fuchs M., John B.V., Acharya C., Sikaroodi M. (2019). Specific Gut and Salivary Microbiota Patterns Are Linked with Different Cognitive Testing Strategies in Minimal Hepatic Encephalopathy. Am. J. Gastroenterol..

[73] Ciocan D., Voican C.S., Wrzosek L., Hugot C., Rainteau D., Humbert L., Cassard A.M., Perlemuter G. (2018). Bile acid homeostasis and intestinal dysbiosis in alcoholic hepatitis. Aliment. Pharmacol. Ther..

[74] Ren Z., Li A., Jiang J., Zhou L., Yu Z., Lu H., Xie H., Chen X., Shao L., Zhang R. (2019). Gut microbiome analysis as a tool towards targeted non-invasive biomarkers for early hepatocellular carcinoma. Gut.

[75] Bajaj J.S., Hylemon P.B., Ridlon J.M., Heuman D.M., Daita K., White M.B., Monteith P., Noble N.A., Sikaroodi M., Gillevet P.M. (2012). Colonic mucosal microbiome differs from stool microbiome in cirrhosis and hepatic encephalopathy and is linked to cognition and inflammation. Am. J. Physiol. Gastrointest. Liver Physiol..

[76] Qin N., Yang F., Li A., Prifti E., Chen Y., Shao L., Guo J., Le Chatelier E., Yao J., Wu L. (2014). Alterations of the human gut microbiome in liver cirrhosis. Nature.

[77] Zhang Z., Zhai H., Geng J., Yu R., Ren H., Fan H., Shi P. (2013). Large-scale survey of gut microbiota associated with MHE Via 16S rRNA-based pyrosequencing. Am. J. Gastroenterol..

[78] Grat M., Holowko W., Wronka K.M., Grat K., Lewandowski Z., Kosinska I., Krasnodebski M., Wasilewicz M., Galecka M., Szachta P. (2015). The relevance of intestinal dysbiosis in liver transplant candidates. Transpl. Infect. Dis..

[79] Tuomisto S., Pessi T., Collin P., Vuento R., Aittoniemi J., Karhunen P.J. (2014). Changes in gut bacterial populations and their translocation into liver and ascites in alcoholic liver cirrhotics. BMC Gastroenterol..

[80] Bajaj J.S., Heuman D.M., Hylemon P.B., Sanyal A.J., White M.B., Monteith P., Noble N.A., Unser A.B., Daita K., Fisher A.R. (2014). Altered profile of human gut microbiome is associated with cirrhosis and its complications. J. Hepatol..

[81] Ahluwalia V., Betrapally N.S., Hylemon P.B., White M.B., Gillevet P.M., Unser A.B., Fagan A., Daita K., Heuman D.M., Zhou H. (2016). Impaired Gut-Liver-Brain Axis in Patients with Cirrhosis. Sci. Rep..

[82] Bajaj J.S., Vargas H.E., Reddy K.R., Lai J.C., O’Leary J.G., Tandon P., Wong F., Mitrani R., White M.B., Kelly M. (2019). Association Between Intestinal Microbiota Collected at Hospital Admission and Outcomes of Patients with Cirrhosis. Clin. Gastroenterol. Hepatol..

[83] Bajaj J.S., Betrapally N.S., Hylemon P.B., Thacker L.R., Daita K., Kang D.J., White M.B., Unser A.B., Fagan A., Gavis E.A. (2015). Gut Microbiota Alterations can predict Hospitalizations in Cirrhosis Independent of Diabetes Mellitus. Sci. Rep..

[84] Albhaisi S., Shamsaddini A., Fagan A., McGeorge S., Sikaroodi M., Gavis E., Patel S., Davis B.C., Acharya C., Sterling R.K. (2021). Gut Microbial Signature of Hepatocellular Cancer in Men with Cirrhosis. Liver Transpl..

[85] Oh T.G., Kim S.M., Caussy C., Fu T., Guo J., Bassirian S., Singh S., Madamba E.V., Bettencourt R., Richards L. (2020). A Universal Gut-Microbiome-Derived Signature Predicts Cirrhosis. Cell Metab..

[86] Bajaj J.S., Thacker L.R., Fagan A., White M.B., Gavis E.A., Hylemon P.B., Brown R., Acharya C., Heuman D.M., Fuchs M. (2018). Gut microbial RNA and DNA analysis predicts hospitalizations in cirrhosis. JCI Insight.

[87] Dubinkina V.B., Tyakht A.V., Odintsova V.Y., Yarygin K.S., Kovarsky B.A., Pavlenko A.V., Ischenko D.S., Popenko A.S., Alexeev D.G., Taraskina A.Y. (2017). Links of gut microbiota composition with alcohol dependence syndrome and alcoholic liver disease. Microbiome.

[88] Caussy C., Tripathi A., Humphrey G., Bassirian S., Singh S., Faulkner C., Bettencourt R., Rizo E., Richards L., Xu Z.Z. (2019). A gut microbiome signature for cirrhosis due to nonalcoholic fatty liver disease. Nat. Commun..

[89] Vilstrup H., Amodio P., Bajaj J., Cordoba J., Ferenci P., Mullen K.D., Weissenborn K., Wong P. (2014). Hepatic encephalopathy in chronic liver disease: 2014 Practice Guideline by the American Association for the Study of Liver Diseases and the European Association for the Study of the Liver. Hepatology.

[90] Patidar K.R., Bajaj J.S. (2015). Covert and Overt Hepatic Encephalopathy: Diagnosis and Management. Clin. Gastroenterol. Hepatol..

[91] Llovet J.M., Zucman-Rossi J., Pikarsky E., Sangro B., Schwartz M., Sherman M., Gores G. (2016). Hepatocellular carcinoma. Nat. Rev. Dis. Primers.

[92] Zeng Y., Chen S., Fu Y., Wu W., Chen T., Chen J., Yang B., Ou Q. (2020). Gut microbiota dysbiosis in patients with hepatitis B virus-induced chronic liver disease covering chronic hepatitis, liver cirrhosis and hepatocellular carcinoma. J. Viral Hepat..

[93] Gerard P. (2013). Metabolism of cholesterol and bile acids by the gut microbiota. Pathogens.

[94] Rinninella E., Raoul P., Cintoni M., Franceschi F., Miggiano G.A.D., Gasbarrini A., Mele M.C. (2019). What is the Healthy Gut Microbiota Composition? A Changing Ecosystem across Age, Environment, Diet, and Diseases. Microorganisms.

[95] Hollister E.B., Gao C., Versalovic J. (2014). Compositional and functional features of the gastrointestinal microbiome and their effects on human health. Gastroenterology.

[96] El Aidy S., van den Bogert B., Kleerebezem M. (2015). The small intestine microbiota, nutritional modulation and relevance for health. Curr. Opin. Biotechnol..

[97] Luo W., Guo S., Zhou Y., Zhao J., Wang M., Sang L., Chang B., Wang B. (2022). Hepatocellular Carcinoma: How the Gut Microbiota Contributes to Pathogenesis, Diagnosis, and Therapy. Front. Microbiol..

[98] Bajaj J.S., Fagan A., Gavis E.A., Mousel T., Gallagher M.L., Puri P., Fuchs M., Davis B.C., Hylemon P.B., Zhou H. (2024). The RIVET RCT: Rifamycin SV MMX improves muscle mass, physical function, and ammonia in cirrhosis and minimal encephalopathy. Hepatol. Commun..

[99] Scarpellini E., Abenavoli L., Cassano V., Rinninella E., Sorge M., Capretti F., Rasetti C., Svegliati Baroni G., Luzza F., Santori P. (2022). The Apparent Asymmetrical Relationship Between Small Bowel Bacterial Overgrowth, Endotoxemia, and Liver Steatosis and Fibrosis in Cirrhotic and Non-Cirrhotic Patients: A Single-Center Pilot Study. Front. Med..

[100] Yokoyama K., Sakamaki A., Takahashi K., Naruse T., Sato C., Kawata Y., Tominaga K., Abe H., Sato H., Tsuchiya A. (2022). Hydrogen-producing small intestinal bacterial overgrowth is associated with hepatic encephalopathy and liver function. PLoS ONE.

[101] Leitner U., Brits A., Xu D., Patil S., Sun J. (2024). Efficacy of probiotics on improvement of health outcomes in cirrhotic liver disease patients: A systematic review and meta-analysis of randomised controlled trials. Eur. J. Pharmacol..

[102] Guo Y., Zhang Y., Gerhard M., Gao J.J., Mejias-Luque R., Zhang L., Vieth M., Ma J.L., Bajbouj M., Suchanek S. (2020). Effect of Helicobacter pylori on gastrointestinal microbiota: A population-based study in Linqu, a high-risk area of gastric cancer. Gut.

[103] Page M.J., McKenzie J.E., Bossuyt P.M., Boutron I., Hoffmann T.C., Mulrow C.D., Shamseer L., Tetzlaff J.M., Akl E.A., Brennan S.E. (2021). The PRISMA 2020 statement: An updated guideline for reporting systematic reviews. BMJ.

