# Supplementary material for: A Systematic Review of Microbiota in Cirrhosis: A Change Towards a More Pathogenic Predisposition
Source: Int J Mol Sci. 2025 Jan 9;26(2):527. doi: 10.3390/ijms26020527 (PMC11765289; doi:10.3390/ijms26020527)
Supplement: Supplementary file 1 [file ijms-26-00527-s001.zip › Table S4.rtf]

	Liver Cirrhosis patients	Chronic Liver Disease with no Cirrhosis (HBV, ALD, NAFLD etc.)	Healthy subjects	Hepatocellular cancer	
East Asia (China, South Korea, Japan)	1553	758	1639	367	
South Asia (India, Taiwan)	84	92	73	29	
America (USA, Canada, Mexico)	3094	72	372	85	
North and central Europe (England, France, Germany, Poland, Finland, Austria)	288	152	243	48	
South Europe and Israel (Greece, Spain, Italy)	233	100	86	21	
Russia	221	72	81		
	5473	1246	2494	550	
Table S4. Studies selected for analysis.
